# Biomechanical role of osteoporosis affects the incidence of adjacent segment disease after percutaneous transforaminal endoscopic discectomy

**DOI:** 10.1186/s13018-019-1166-1

**Published:** 2019-05-14

**Authors:** Jingchi Li, Wenqiang Xu, Xiaoyu Zhang, Zhipeng Xi, Lin Xie

**Affiliations:** 1 Department of Orthopedic Surgery, Jiangsu Province Hospital on Integration of Chinese and Western Medicine, 100th Shizi Street, Nanjing, 210028 Jiangsu People’s Republic of China; 2 Department of Spine Surgery, Third Clinical Medical College of Nanjing University of Chinese Medicine, Nanjing, 210028 Jiangsu People’s Republic of China

**Keywords:** Adjacent segment diseases, Percutaneous transforaminal endoscopic discectomy, Osteoporosis, Bone mineral density, Finite element method

## Abstract

**Study design:**

Variation in the biomechanical characteristics of intervertebral discs adjacent to the segment disc after undergoing percutaneous transforaminal endoscopic discectomy (PTED) in models with normal and abnormal bone mineral density (BMD) was estimated using the finite element method.

**Objective:**

The study investigated the change in the incidence of adjacent segment disease (ASD) after PTED in patients without and with osteoporosis.

**Backgrounds:**

PTED has been widely used for treating lumbar disc herniation (LDH); changes in BMD will affect biomechanical characteristics, possibly leading to changes in the incidence of ASD after PTED. However, this issue remains largely unclear.

**Methods:**

A non-linear, lumbosacral finite element model was reconstructed based on imaging data and validated using compared values computed by the current model from published and well-validated, in vitro biomechanical experiment studies. Corresponding PTED models with normal and abnormal BMDs were also reconstructed. Shear and von Mises stresses on the annulus fibrosis, the von Mises stress on the endplates in L5–S1 segment discs, and the total deformation of current lumbosacral models were computed in different body positions by changing loading conditions, including flexion, extension, left and right lateral bending, and axial rotation.

**Results:**

In most loading conditions, biomechanical characteristics of the lumbosacral segment discs with normal BMDs after PTED slightly increased. However, in the PTED model with osteoporosis, most of the biomechanical characteristics dramatically increased.

**Conclusion:**

Osteoporosis leads to the deterioration of biomechanical characteristics in the adjacent segment disc after PTED; this variation may also result in an increase in the incidence of ASD. However, further studies on the interactions between pathological changes are warranted.

## Key points

PTED can lead to a slight deterioration in the biomechanical characteristics of adjacent segment discs, which are significantly intensified in an osteoporosis model.

Because women are more likely to suffer from osteoporosis, intensified postoperative biomechanical deterioration in patients with osteoporosis can be seen as a credible reason for the higher risk of ASD after PTED among elderly women.

The interaction of risk factors of ASD was not estimated in the current study, warranting further research.

## Introduction

ASD is a series of postoperative complications, including types of symptoms as low back pain (LBP) and low limb pain or numbness [1, 2]. The etiological factors and demographic characteristics of ASD have been widely discussed. The risk factors concerning demographic data, such as senility, female sex, and obesity, as well as etiological triggers such as severe stress concentration, hypermotility, and preexisting degenerative changes in adjacent segment discs have been mentioned [1, 3–6].

Biomechanical deterioration and resulting injury to spine structures can be seen as the most significant triggers for the development of disc degeneration change and which is one the most important reasons of ASD [1–3, 7–10]. While discussing the risk factors for ASD, demographic characteristics are always assumed to be defined by some biomechanical pathogenesis. For instance, overweight patients suffer from excessive pressure in the lumbar spine, which may accelerate disc degeneration. This can explain why patients with higher body mass indices (BMI) are more prone to ASD [1, 3, 11]. And multiple studies have revealed that the incidence of ASD is high in senile patients and that preexisting disc degeneration changes is a vital risk factor of ASD [1–3]. Considering that disc degeneration becomes more pronounced with increasing age, we believe that senile patients always suffer from ASD because of preexisting degenerations [5, 6].

A part of patients suffer from lumbar disc herniation (LDH) accompanied with osteoporosis, and women, especially elderly women, are more likely to suffer from osteoporosis [12]. As mentioned earlier, women are more vulnerable to ASDs [3, 5]; therefore, logically, osteoporosis should be implicated in the occurrence of ASDs. Nevertheless, the clinical review by Wang [1] indicated that the risk of ASD can be altered by changing the BMD and that the most important indicator of osteoporosis was not statistically different in patients with or without ASD. However, a review by Park et al. [3] highlighted that osteoporosis can be deemed as an independent predictor of ASD.

Meanwhile, some published literature revealed that changes in BMD exert a vital impact on the biomechanical characteristics of the spine as well as affect the incidence of postoperative complications [13–15]. For biomechanical deterioration is important in the development of ASD, exploration of biomechanical mechanism is of great significance for the investigation of the pathogenesis of ASD, and different surgical methods have been computed by finite element methods (FEM) to estimate if which could decrease the risk of ASD or not [16–18]. However, no published studies illustrated this issue after PTED, although it became increasingly popular for treating LDH. In consideration of this, the ASD will lead to a poor prognosis for patients [3]. Investigations concerning risks of ASD in PTED patients are essential.

We hypothesized that this change affects the incidence of ASD, and no statistically significant conclusion from some clinical report may origin from relatively small sample size or short follow-up period. In addition, to the best of our knowledge, no published English biomechanical study has focused on whether osteoporosis affects the incidence of ASD after PTED. To test this hypothesis and to clarify the abovementioned issue, a lumbosacral three-dimensional model was reconstructed and validated, and corresponding PTED models with and without osteoporosis were reconstructed to determine whether changes in BMD influence shear and the von Mises stresses on the annulus fibrosis, the von Mises stress on the endplates, and the total deformation of the lumbosacral spine. All the characteristics were assessed in the L5–S1 segment, except for the total deformation of spine model, owing to the high incidence of disc degenerative diseases in this segment [7].

## Material and methods

### Reconstruction of a complete spine model

A 3D lumbosacral model was reconstructed based on the imaging data obtained from an adult male volunteer (L3–S1, three segments of the intervertebral disc (IVD), six facet joints, and six ligaments) without any history of lumbar diseases using computed tomography (CT). The structures in the current model could not be clearly differentiated using CT and have therefore been constructed based on our anatomical observations [13, 19]. In the current study, the vertebra included a cortical shell (thickness 0.8 mm), a trabecular core, superior and inferior endplates (thickness: 0.8 mm), and posterior structures. IVD comprised the annulus fibrosis and inner nucleus, which occupied 44% of the cross-sectional area of the disc, whose location was slightly backward to the disc center, and comprised facet joints including capsule and facet cartilage surfaces (0.25 mm) and ligaments including the anterior longitudinal ligament, posterior longitudinal ligament, ligamentum flavum, intertransverse ligament, interspinous ligament, and supraspinous ligament [18, 20, 21].

### Reconstruction of PTED and osteoporosis models

**Fig. 1:**
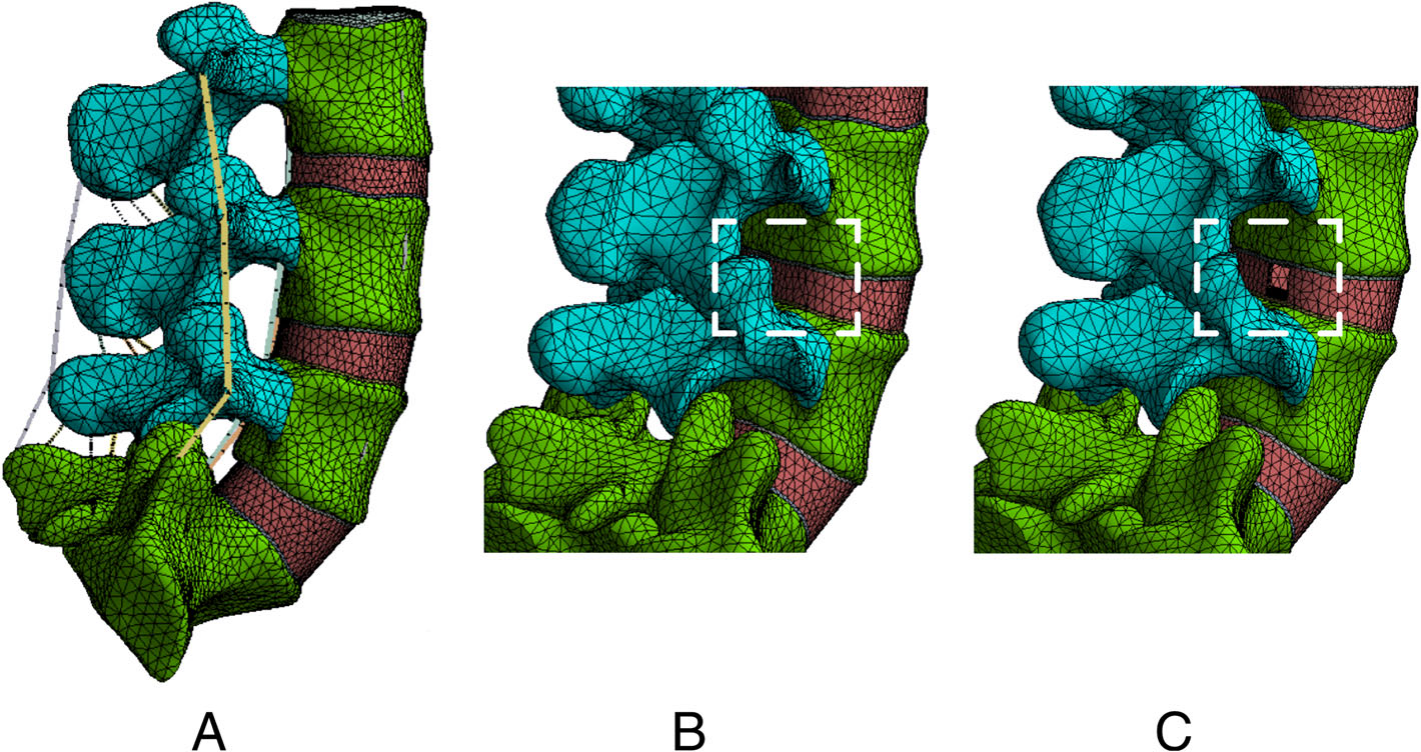
Diagrams of model reconstruction (all ligaments have been hidden in the schematic diagrams for PTED in order to display the surgical procedures clearly. And osteoporosis has been omitted for reconstruction, of which only adjusted material properties without the change of structures are shown) **a** Schematic diagram of the complete lumbosacral model. **b** Model 1: contact model. **c** Model 2: PTED model

**Fig. 2:**
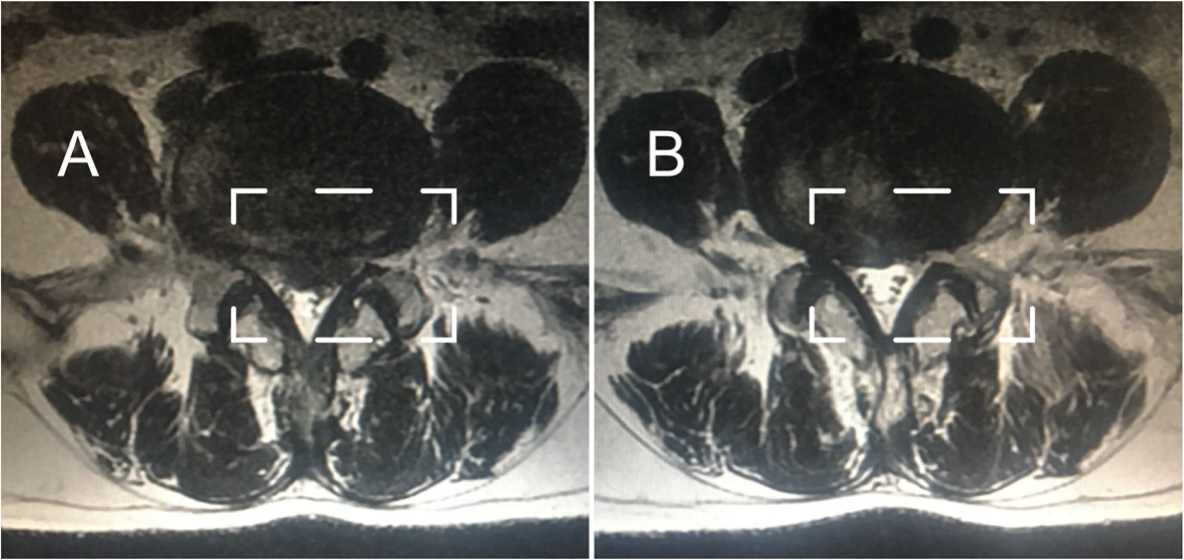
The MRI of patients subjected to PTED with different grades of facetectomy. **a** Preoperative imagine data. **b** Postoperative imagine data

The L4–L5 segment, which is one of the most common sites of LDH, was selected for the simulation of PTED. PTED was simulated on the right side. To imitate tears in the annulus fibrosis, a 5-mm incision was made and to represent discectomy, the nucleus in this segment was deleted [22, 23]. Moreover, facetectomy and ligamentum flavum excision are inevitable in PTED, and therefore, sufficient space was created to insert instruments and remove the nucleus. In this, one third of each of these structures were removed [21, 22, 24, 25]. The schematic diagram of current models is shown in Fig. 1, and the magnetic resonance imaging (MRI) date of patients subjected to PTED is shown in Fig. 2.

The reconstruction of osteoporosis FEM models has been described in the scientific literature. The Young modulus in different parts of the current model has been changed, but morphological features remained the same [14, 15, 17]. The Young modulus for cortical, endplate, and posterior structures decreased to 67% of its original value. The Young modulus in cancellous bone decreased to 34% [13–15].

### Boundary and loading conditions

Boundary and loading conditions were uniformly applied in the three models in this study. To simulate the complicated spinal motion, five different loading conditions, including flexion, extension, left and right bending, and axial torsion, were applied by setting a 10-Nm moment on the superior surface of the lumbosacral model and a 800-N vertical compressive force in the basic loading condition [9, 17].

To reduce the computed error, tetrahedral elements were selected to fill the current model because they seemed suitable for models with complex surfaces; moreover, mesh refinement was set in distortion areas. In current models, the contact type was set as “bounded”; however, for facet surfaces, it was set as “frictionless” [9, 17]. Regarding the material properties, the vertebra and facet cartilages were defined as isotropic, homogeneous materials; the Mooney–Rivlin hyper-elastic material was set in the annulus fibrosis; and the nucleus was defined as an incompressible semifluid material. Moreover, “tension-only” cable elements were used for defining the ligaments (Tables 1 and 2) [13, 18]. In addition, for simplification, the complete lumbosacral model was defined as model 1, the model that underwent PTED with normal BMD as model 2, and the model with osteoporosis as model 3.

**Table 1:** The material properties of the current finite element models

Components	Young’s modulus (osteoporosis)	Poisson’s ratio	Cross-sectional area (mm^2^)
Cortical	12000 (8070)	0.3	/
Cancellous	100 (34)	0.2	/
Posterior elements	3500 (2345)	0.25	/
Endplate	1000 (670)	0.4	/
Cartilage	10	0.4	/
Capsular	26	0.3	67.5
ALL	20	0.3	60
PLL	70	0.3	21
LF	50	0.3	60
ITL	50	0.3	10
ISL	28	0.3	40
SSL	28	0.3	30

Material properties of the osteoporosis model are shown in brackets

*ALL* anterior longitudinal ligament, *PLL* posterior longitudinal ligament, *LF* ligamentum flavum, *ISL* interspinous ligament, *SSL* supraspinal ligament, *ITL* intertransverse ligament

**Table 2:** The material properties of intervertebral discs

Annulus		Nucleus	
C1 (Mpa)	C2 (Mpa)	Young’s modulus (Mpa)	Poisson’s ratio
0.2	0.05	1	0.499

## Results

### Model validation

The intradiscal pressure in the L4-L5 segment was validated by comparing with a previously published, well-validated in vitro biomechanical study, and the disc compression values were validated by comparing with another in vitro research. To calculate the intradiscal pressure, 300, 1000, and 2000 N of vertical compressive pressure was applied on the superior surface of model 1 and 1200 N of vertical compressive pressure was applied to assess disc compression [22, 25].

**Fig. 3:**
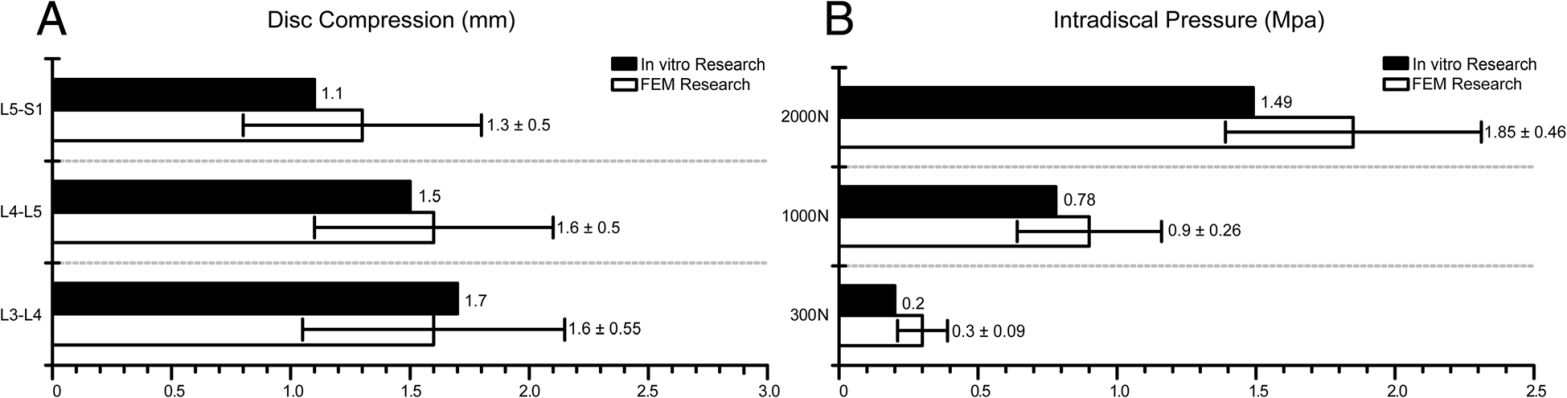
Validation of the FEM model. **a** The disc compression. **b** The intradiscal pressure

At 300, 1000, and 2000 N of vertical compressive force, the intradiscal pressure in our model was 0.2, 0.78, and 1.49 Mpa and that previously reportedly was 0.3 ± 0.09, 0.9 ± 0.26, and 1.85 ± 0.46 Mpa, respectively [22]. Disc compression values in the L3–S1 segment in the current model were 1.7, 1.5, and 1.1 mm and those from a well-validated research were 1.6 ± 0.55, 1.6 ± 0.5, and 1.3 ± 0.5 mm in L3–L4, L4–L5, and L5–S1 segment disc, respectively [25]. Thus, the values in the present study were within one standard deviation from those reported in the well-validated studies except the intradiscal pressure under 300-N compressive pressure (Fig. 3). Considering the only exception listed immediately below the standard deviation and equaling to the lower value in the original data, we believe that the current model can make a credible representation of the lumbosacral spine in actual body positions [22].

### Biomechanical characteristics variation

Notably, the trend in biomechanical characteristics increased in model 2 in most loading conditions, except for the von Mises stress in the annulus in extension condition and when the endplates were in left lateral bending. Meanwhile, except for the shear stress of the annulus and the von Mises stress in the endplates in the flexion condition, most of the biomechanical characteristics increased by < 5%.

**Fig. 4:**
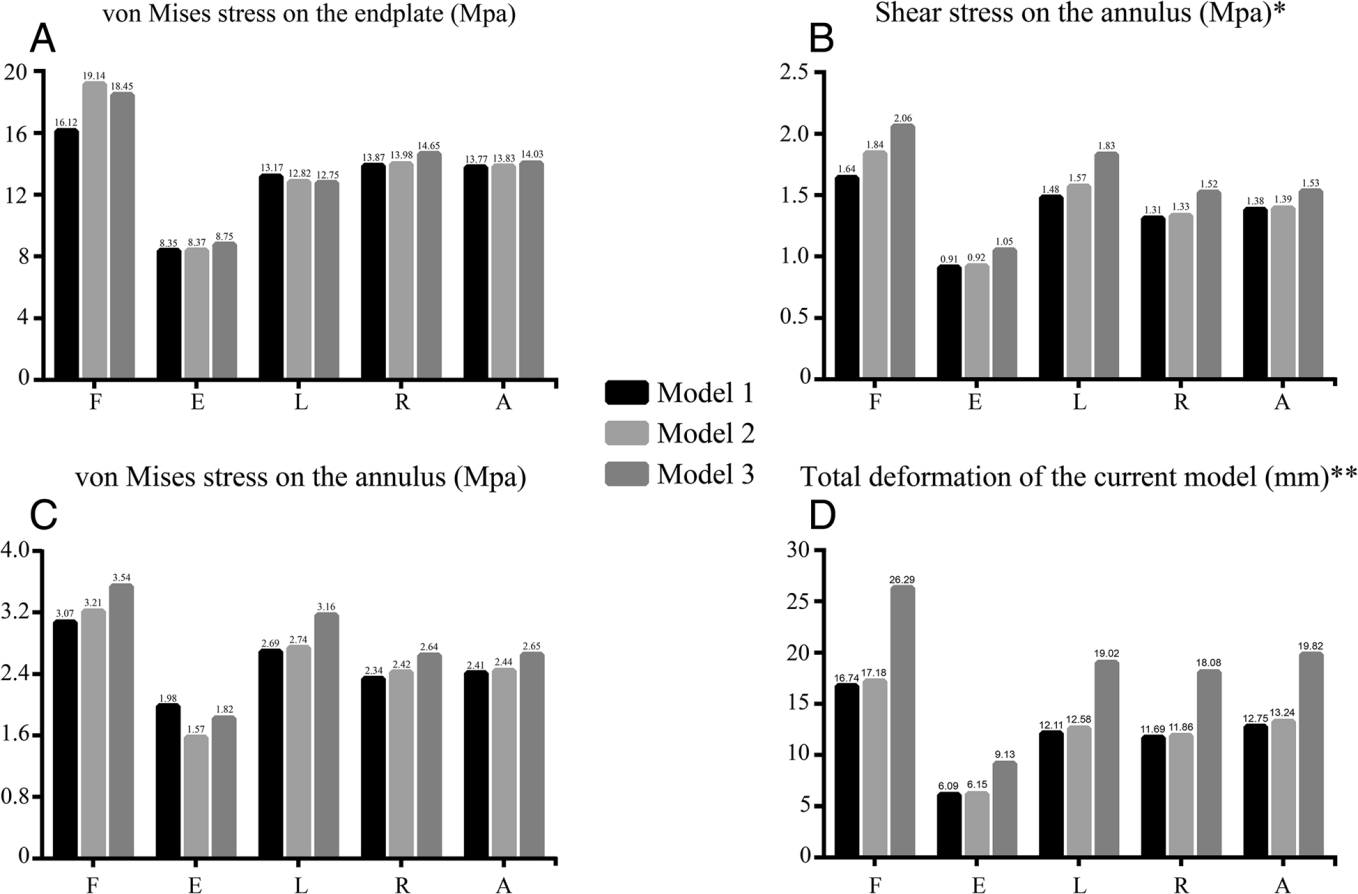
**a**–**d** Biomechanical changes in different models. F, Flexion; E, extension; L, left lateral bending; R, right lateral bending; A, Axial rotation. Model 1: contact model. Model 2: PTED model with normal bone mineral density. Model 3: PTED model with osteoporosis

However, this trend dramatically changed with respect to the differences between model 3 and the other two models. Compared with that in model 2, the von Mises stress in endplates was lower in flexion and left lateral bending condition in model 3, and this value in the annulus in extension condition increased in model 3 but was lower than that in model 1. In addition, all the other biomechanical characteristics increased dramatically in model 3 as compared with those in models 1 and 2 (Fig. 4).

## Discussion

The risk factors of ASD have been widely investigated, and the demographic results from published literature indicate that old age, high BMI, and female sex increase the risk of incidence of ASD [1, 3, 4, 11]. Meanwhile, hypermotility of the lumbar spine, abnormal stress concentration, and preexisting degenerative changes in the adjacent discs can also trigger ASD [5, 6].

The aberrant distribution of stress and the resulting injury of disc structures have been regarded as the principal factors of disc degeneration and have been proven to be the primary risk factors for ASD [3, 7, 8]. Therefore, we believe that the high risk of the incidence of ASD in a specific population may be connected with a biomechanical cause. Moreover, our hypothesis seems reliable considering that a high incidence of ASD in senile patients may root from multiple segment degenerations in lumbar discs before surgery and that in obese patients may be connected to the greater load on the spine [26].

However, it is unclear as to why women are more vulnerable to ASD, considering that the biomechanical factors that are common in women and are related to ASD have not been clearly estimated. Published studies have illustrated that higher expression of estrogen receptor in the facet cartilage is connected to degenerative spondylolisthesis (a typical manifestation of lumbar instability) [27, 28] and is an important reason for ASD [1, 8, 23]. This seems to be a reasonable explanation for the relatively higher risk of ASD in women. However, considering the decisive role of biomechanical deterioration in the pathological changes of ASD [1, 3], an investigation of the relationship between biomechanical factors common to females and the pathogenesis of ASD is still necessary.

Women, particularly elderly women, are susceptible to osteoporosis. As per the epidemiological investigation report by Ha [12], women are approximately three times more prone to osteoporosis than men. Considering that Chinese and Korean studies focus on Asian populations, this data can be used as a reference in studies including Chinese population, indicating that the relationship between the higher risk of ASD in women may be connected with a higher incidence of osteoporosis. Published studies have reported that osteoporosis may increase the risk of disc degeneration in the lumbar spine by adversely changing the loading pattern and intradiscal pressure [29, 30]. We therefore hypothesize that osteoporosis is a key reason for the relatively higher risk of ASD in women.

LDH is one of the most common disc degenerative diseases that is widely treated by surgery, and investigation record-related risk factors of ASD have always focused on the fusion methods rather than on PTED although it has been widely used in the past decade [2, 31]. For the abovementioned reasons, we believe study concerning the biomechanical influence of the alteration of BMD for the ASD risk for PTED patients is clinically important. Moreover, to investigate this issue, a lumbosacral finite element model was reconstructed to determine the change in shear and the von Mises stresses on the annulus fibrosis, the von Mises stress on the endplates, and the total deformation of models with and without osteoporosis undergoing PTED.

Stress concentration typically results from abnormal stress distribution, which eventually leads to the formation of injury structures in IVDs [8]. The endplates play an irreplaceable role during the process of loading conditions; therefore, damage to the endplates can result in various issues. For instance, stress concentration on the trabecular bone may result in microfractures, disrupting the nutrient pathway of the IVDs. Considering that IVD is an avascular structure, the disruption of nutrition may severely accelerate the process of disc degeneration [32]. The von Mises stress in endplates increased in most loading conditions in the osteoporosis model, which may increase the incidence of ASD associated with changes in the endplates.

**Fig. 5:**
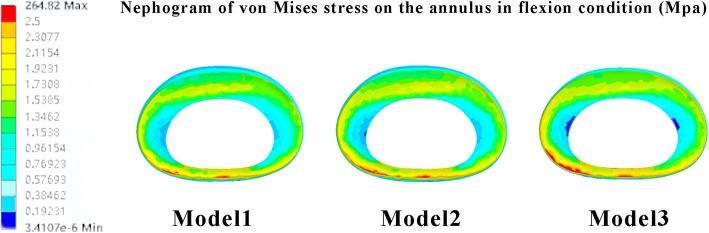
Nephogram of von Mises stress on the annulus in flexion condition. Model 1: contact model. Model 2: PTED model with normal bone mineral density. Model 3: PTED model with osteoporosis

**Fig. 6:**
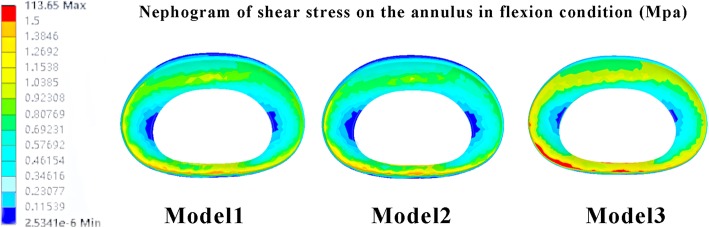
Nephogram of shear stress on the annulus in flexion condition. Model 1: contact model. Model 2: PTED model with normal bone mineral density. Model 3: PTED model with osteoporosis

Abnormal stress concentration on the annulus can result in tears. This trend is connected with the generation of ASD [3, 7]. For example, the von Mises stress concentration is connected with the circumferential tears and increased shear stress is strongly linked to radical tears that can lead to discogenic LBP and increase the risk of LDH in adjacent segment discs, which are two typical manifestations of ASD [9, 21, 33]. Moreover, endplate injury intensifies abnormal stress distribution in the annulus and aggravates the incurred damage [7, 34]. As presented in our FEM results, the trend of stress concentration can be observed in the PTED model of osteoporosis. As mentioned in the theories presented above, the risk of annulus tears and subsequent ASD may increase in osteoporosis patients (Figs. 5 and 6).

Considering that the deformations of the spine mainly occur in IVDs rather than in vertebral bodies, we believe that the increase in total deformation in the current model is a good representation of the hypermotility of the adjacent IVDs [3]. The hypermotility of IVDs has been proven to trigger LBP, a typical symptom of ASD [31, 35]. Moreover, a change in this index is an important and independent predictor of ASD, which could accelerate the process of disc degeneration [3]. Therefore, increase in the total deformation in the osteoporosis model may predict an increasing trend of the incidence of ASD in patients with osteoporosis after PTED.

In a summary, it can be concluded that osteoporosis may be an independent risk factor of ASD for patients undergoing PTED, which may be the cause of the high incidence of ASD in women.

Nevertheless, some limitations in the current study still exist. For instance, several pathological changes, such as osteoporosis, osteoarthritis of facet cartilage, and multiple-segment degeneration, can be observed in PTED patients (especially elderly patients). The interaction of these pathological changes may strongly impact the risk of ASD and affect the credibility of our conclusion [9, 15, 32]. These issues require further investigation. Hence, the existing conclusion may be inaccurate or only applicable to patients without degenerative changes in other segments. Besides, the model constructed in this study omitted soft tissues around the spine. As a result, the current FEM data pertain only to ideal cases. All of these factors should be explored in future studies.

## Conclusion

Osteoporosis may be regarded as an independence risk factor of ASD after PTED, and this association can explain the phenomenon of women being more likely to suffer from ASD.

## Abbreviations

ASD: Adjacent segment disease
BMD: Bone mineral density
BMI: Body mass indices
CT: Computed tomography
FEM: Finite element method
IVD: Intervertebral disc
LBP: Low back pain
LDH: Lumbar disc herniation
MRI: Magnetic resonance imaging
PTED: Percutaneous transforaminal endoscopic discectomy
